# Short- and long-term mental health consequences of work-to-family conflict: a 15-year prospective, population-based cohort study

**DOI:** 10.1192/j.eurpsy.2026.10238

**Published:** 2026-07-31

**Authors:** Y. Ni, R. Long, N. Liu, C. S. Wong, W. W. Mak, S. Manuo, M. Y. Ni

**Affiliations:** 1 School of Public Health, The University of Hong Kong; 2 Department of Psychology, The Chinese University of Hong Kong, Hong Kong, Hong Kong; 3 Faculty of Social Sciences, Tampere University, Tampere, Finland

## Abstract

**Introduction:**

Work-family conflict is a widespread issue in modern workforces. However, longitudinal evidence on its mental health impacts remains mixed and inconclusive. While research on non-Western populations is growing, significant gaps remain, especially regarding anxiety and factors that influence the conflict’s effects on mental health.

**Objectives:**

To examine the short- and long-term contributions of work-to-family conflict (WFC) to mental health and identify factors that may modify the effects of WFC on mental health.

**Methods:**

The study was built on the FAMILY Cohort, a large, population-based cohort study in Hong Kong. Two household visits were completed in 2009-11 (Wave 1) and 2011-14 (Wave 2) and a telephone survey was completed in 2023-24 (Wave 3). Mental health outcomes included depressive symptoms (score ≥5) and probable depression (score ≥10), assessed using the Patient Health Questionnaire-9, as well as anxiety symptoms (score ≥5) and probable anxiety (score ≥10), assessed using the Generalized Anxiety Disorder Scale. We also analysed PHQ-9 and GAD-7 scores as continuous outcomes. Moderators included sex, socioeconomic status (SES), family structure, social support from various sources (e.g., domestic helper, supervisor, co-worker, organisational level), and work-family benefits.

**Results:**

The analytic samples comprised 6,056 participants for the short-term (Wave 1 to Wave 2) and 1,061 participants for the long-term (Wave 1 to Wave 3) mental health consequences. Higher baseline WFC scores were associated with short-term depressive symptom scores (incident rate ratio (IRR): 1.04, 95% CI: 1.01-1.07) and long-term anxiety symptom scores (IRR: 1.10, 1.03-1.17) in the fully adjusted models. No significant associations were identified for binary outcomes in the overall sample. Further analyses revealed that these associations were significant among men, specifically, for short-term depressive symptoms (odds ratio (OR): 1.16, 1.05-1.28) and long-term probable anxiety (OR: 1.44, 1.10-1.88), but not among women. The association with long-term anxiety symptoms was strongest among unmarried parents with children aged <10 (IRR: 1.33, 1.14-1.54). Access to childcare support from a domestic helper and the availability and utilisation of work-family policies appeared to buffer the long-term negative effects of WFC on anxiety outcomes.

**Conclusions:**

Our findings provide compelling evidence of the mental health consequences of WFC, revealing gender-specific patterns and emphasising the vital role of domestic helper and work-family policies in balancing work and family demands and promoting mental health in working adults in Hong Kong. The relevance of our findings extends to other Chinese and Asian cities with similar socio-cultural contexts, as well as rapidly developing and high-income cities globally, where WFC is an escalating challenge.

**Disclosure of Interest:**

None Declared

